# Hypophosphatemia in Intensive Care Unit Canine Patients: Occurrence and Association With Mortality and Duration of Hospitalization

**DOI:** 10.1111/vcp.70013

**Published:** 2025-06-02

**Authors:** Ioannis L. Oikonomidis, Paul Rees, Jorge Hernando Sanz, Glynn Woods

**Affiliations:** ^1^ Institute of Infection, Veterinary and Ecological Studies University of Liverpool Neston UK; ^2^ Royal (Dick) School of Veterinary Studies and the Roslin Institute, the University of Edinburgh Roslin UK

**Keywords:** critical illness, dog, ICU, phosphorus, survival

## Abstract

**Background:**

Hypophosphatemia is commonly observed in unselected human intensive care unit (ICU) patients, and it has been associated, although inconsistently, with worse outcomes and longer duration of hospitalization (DOH). The incidence of hypophosphatemia and its association with mortality and DOH in ICU canine patients is unknown.

**Objectives:**

The aims of this study were to determine the occurrence of hypophosphatemia in unselected ICU canine patients and its association with mortality and DOH.

**Methods:**

The medical records of all dogs admitted to the Teaching Hospital ICU between January 2019 and December 2023 were retrospectively reviewed. Dogs with hypophosphatemia (serum phosphate < 0.9 mmol/L) were identified. For every hypophosphataemic dog included in this study, two age‐matched control, non‐hypophosphataemic dogs, closely admitted to the ICU in time, were included.

**Results:**

In total, 3233 medical records were reviewed. Hypophosphatemia was noted in ≥ 1 day of hospitalization in 32 dogs (0.99%). The age‐matched case and control groups had a median (range) age of 8.0 (1.0–14.0) years. The survival to discharge rates of both hypophosphataemic and control groups were 78% (25/32 and 50/64, respectively), and their DOH (median, 3.5 days; range 1.0–9.0 and median 3.0 days; range 1.0–25.0, respectively) were not significantly different (*p* = 0.557). Serum phosphate concentration was not correlated with the DOH (*p* = 0.649).

**Conclusions:**

Hypophosphatemia was noted in only 1% in this canine ICU patient population and was not associated with the survival to discharge and DOH.

## 1 Introduction

Phosphorus is the major intracellular anion in the body and is of paramount importance for the maintenance of cellular membrane integrity and normal bone and dental matrix, energy stores, metabolic processes, messenger systems, regulation of tissue oxygenation, and urinary buffering capacity. The terms phosphorus and phosphate are typically used interchangeably, although they do not describe the same molecule. Phosphorus refers to the chemical element, and phosphate refers to molecules containing PO_4_^3−^, which is how phosphorus is present in the body [1]. Therefore, in this manuscript, the accurate term ‘phosphate’ will be used. In plasma, phosphate exists in both organic and inorganic forms, but only the latter is measured in laboratories and reported (Pi). The inorganic plasma phosphate is present in three forms: approximately 10% is bound to plasma proteins, and the rest is free (55%–85%) or bound to small cations, such as calcium and magnesium (5%–35%).

In general, hypophosphatemia develops due to decreased gastrointestinal absorption (e.g., anorexia, malnutrition, or malabsorption), increased renal excretion (e.g., increased diuresis), or extracellular to intracellular fluid shift (e.g., metabolic alkalosis, refeeding syndrome or insulin therapy). The clinical signs of mild hypophosphatemia are usually vague and include generalized weakness, anorexia, muscle pain, tremors, ataxia, nausea, and vomiting. Severe hypophosphatemia might be associated with life‐threatening signs, such as intravascular hemolysis, acute respiratory failure, cardiac arrhythmias, seizures, and coma.

Hypophosphatemia has an incidence ranging from 15.4% to 53.3% in unselected human intensive care unit (ICU) patients and has been associated with mortality in such patients, with the prevalence ranging from 15.4% to 53.3% [2, 3, 4, 5, 6], although some did not demonstrate that hypophosphatemia is an independent risk factor for mortality [2, 4]. A systematic review and meta‐analysis concluded that in ICU human patients, hypophosphatemia is not an independent outcome predictor but is associated with a longer duration of hospitalization [7].

To the best of our knowledge, the incidence of hypophosphatemia in ICU canine patients and its association with mortality and duration of hospitalization has not been studied. Therefore, the aims of this study were to determine the incidence of hypophosphatemia in unselected canine ICU patients and its association with the outcome and duration of hospitalization. We hypothesized that in ICU canine patients, the association of hypophosphatemia with mortality and longer duration of hospitalization would be similar to that seen in human ICU patients.

## 2 Materials and Methods

The present study was approved by the Veterinary Ethical Review Committee of the Veterinary School (reference number: 63.24). The electronic medical records of all dogs admitted to the ICU of a small animal teaching hospital (Royal (Dick) School of Veterinary Studies and The Roslin Institute, The University of Edinburgh, Roslin, UK), between January 2019 and December 2023 were retrospectively reviewed, and dogs with hypophosphatemia (serum phosphate concentration < 0.9 mmol/L) were identified. Serum phosphate was measured in all dogs using the ammonium molybdate colorimetric method by spectrophotometry (analyzer AU480, Beckman Coulter, UK). Dogs with incomplete medical records and those readmitted were excluded. An age‐matched control group of dogs without hypophosphatemia was generated from the same ICU canine population. Specifically, for every dog with hypophosphatemia (case), two dogs without hypophosphatemia (controls) were assigned. The two controls represented the temporally closest admissions before or following the admission of the case that had serum phosphate measured using the same biochemistry analyzer. Data were collected from the medical records, including signalment, clinical and laboratory findings, diagnosis, survival to discharge from the hospital, and the duration of hospitalization. Non‐survivors included dogs that died or were euthanized due to grave prognosis during hospitalization. The body condition was scored using a nine‐point scale according to WSAVA guidelines.

The data distribution pattern was determined by the Shapiro–Wilk test. Categorical variable frequencies were compared between groups using the chi‐square test. Continuous variables were compared between groups using the Wilcoxon rank sum test. Correlations between two variables were determined by Spearman's correlation test. Statistical analyses were performed using the statistical language R (R Foundation for Statistical Computing, Austria).

## 3 Results

During the 5‐year study period, hypophosphatemia was recorded in 32 of 3233 dogs (0.99%) admitted to the ICU, in ≥ 1 day of hospitalization, of which 31 dogs were hypophosphatemic upon admission, while 1 developed hypophosphatemia on Day 3 of hospitalization. The median (range) age of the hypophosphatemic dogs was 8.0 (1.0–14.0) years. The group included 6 entire males, 15 castrated males, and 11 spayed females. The age‐matched control group included 64 dogs (10 entire males, 24 castrated males, 7 entire females, and 23 spayed females). There was no statistically significant group difference in the sex distribution (*χ* ^2^ = 0.900, *p* = 0.343). None of the hypophosphatemic dogs was being administered total parenteral nutrition. The diagnoses of the 32 hypophosphatemic dogs are presented in Table 1.

**TABLE 1:** Diagnoses of the 32 hypophosphatemic dogs admitted to the ICU of a veterinary teaching hospital (Royal (Dick) School of Veterinary Studies and The Roslin Institute, The University of Edinburgh, Roslin, UK) between January 2019 and December 2023.

Diagnosis	*n* (%)
Aspiration pneumonia	3 (9%)
Intervertebral disc disease	3 (9%)
Diabetes mellitus	2 (6%)
Pancreatitis	2 (6%)
3rd degree atrioventricular block	1 (3%)
5‐fluoruracil toxicity	1 (3%)
Acute hepatitis	1 (3%)
Acute kidney injury	1 (3%)
Aortic thromboembolism	1 (3%)
Bronchial foreign body	1 (3%)
Dietary indiscretion	1 (3%)
Gastric dilatation and volvulus	1 (3%)
Haemorrhagic gastroenteritis	1 (3%)
Hepatic encephalopathy due to extrahepatic portosystemic shunt	1 (3%)
Idiopathic epilepsy	1 (3%)
Pneumothorax	1 (3%)
Primary hyperparathyroidism	1 (3%)
Ruptured gall bladder mucocele	1 (3%)
Upper respiratory tract obstruction	1 (3%)
Septic peritonitis	1 (3%)
Thyroid carcinoma	1 (3%)
Trauma‐induced seizure	1 (3%)
Dogs diagnosed with > 1 condition – Pneumonia and upper airway disease – Hypoadrenocorticism, chronic enteropathy and pancreatitis – Primary hyperparathyroidism, laryngeal paralysis and hypoadrenocorticism – Wells' syndrome and aspiration pneumonia	4 (13%)

Phosphate concentration was significantly (*p* < 0.001) lower in the study group (median, 0.70 mmol/L; range 0.60–0.80 mmol/L) compared to the control group (median, 1.48 mmol/L; range, 0.90–5.20 mmol/L). The median (range) body condition was not significantly different (*p* = 0.958) between the case group (4 [3–7]) and the control group (4.5 [2–8]). A total of 25 out of 32 (78.1%) hypophosphatemic dogs and 50/64 (78.1%) controls survived to discharge. The median duration of hospitalization was not significantly different (*p* = 0.557) between the case group (3.5 [1–9] days) and the control group (3.0 [1–25] days). When only the survivors were included in the comparison, the median duration of hospitalization was still not significantly different (*p* = 0.573) between the case group (4.0 [1–9] days) and the control group (4.0 [1–25] days). Serum phosphate concentration was not significantly correlated with the duration of hospitalization (rho = −0.047, *p* = 0.649).

## 4 Discussion

To our knowledge, this is the first study investigating the incidence of hypophosphatemia in dogs admitted to the ICU and its association with the outcome and duration of hospitalization. The incidence of hypophosphatemia was only 1% in our unselected canine ICU patients. In this study of unselected canine ICU patients, hypophosphatemia was not associated with survival to discharge and the duration of hospitalization.

The incidence of hypophosphatemia in this cohort was only 1%, in sharp contrast with findings reported in unselected human ICU patients, ranging from 15.4% to as high as 53.3% [2, 3, 4, 5, 6, 8] The reasons for this difference are uncertain. Modern pet food often contains considerably higher phosphorus concentrations than the requirement [9, 10], and dietary phosphorus in pet foods might be present in highly soluble forms [9]. This, combined with the efficient intestinal phosphate absorption in dogs [11], might partially account for the low incidence of hypophosphatemia in this cohort compared to findings in ICU human patients. Additionally, diseases commonly associated with ICU admission in humans, such as cardiovascular diseases, hypertension, chronic obstructive pulmonary disease, and diabetes mellitus, are considerably less frequent in dogs [2, 6, 12]. These differences in the prevalence of diseases associated with ICU admissions between dogs and humans may also be a cause of the different prevalence of hypophosphatemia.

In all but one case in our study, hypophosphatemia was observed at admission, with one case developing hypophosphatemia on the third day of hospitalization. Although hypophosphatemia can occur at any time during the stay of human patients in the ICU, it most commonly develops during the first 3–5 days of hospitalization [3, 4]. It has been suggested that the etiology of this early‐onset hypophosphatemia may differ from that developing during prolonged ICU hospitalization, where malnutrition, vitamin D deficiency, and redistribution between the extracellular and intracellular fluids appear to be the predominant factors [3]. Bech et al. (2013) investigated the etiology of early‐onset hypophosphatemia in ICU human patients and found that increased renal phosphate loss was the underlying cause in 80% of cases [3]. Interestingly, this increased renal loss was not associated with the actions of major phosphaturic hormones like PTH, parathyroid hormone‐related peptide (PTHrp), calcitonin, or FGF‐23 [3]. The authors suggested that the responsible factor for increased phosphate renal loss could be tumor necrosis factor α (TNF‐α), which has been shown to have phosphaturic actions [13]; however, the role of TNF‐α has not been specifically investigated in the ICU setting.

In our study, the outcomes of hypophosphatemic dogs were not different compared with the control group. Hypophosphatemia was initially associated with worse outcomes in surgical ICU human patients [14], and two recent studies found hypophosphatemia to be an independent predictor of mortality in unselected human ICU patients [6, 15]. However, other studies, including two with large sample sizes, indicated that hypophosphatemia may be associated with the severity of illness, but it is not an independent predictor of outcome [2, 4, 5], findings that were confirmed by a recent metanalysis [7]. This aligns with our results, which indicate that hypophosphatemia is not associated with worse outcomes in ICU canine patients. On the other hand, the duration of hospitalization was found to be significantly longer in unselected hypophosphatemic human ICU patients compared to unselected human ICU patients with serum phosphate concentration within the reference interval [4, 5, 6]. In our study, the duration of hospitalization was not significantly different between the hypophosphatemic and control dogs. Overall, hypophosphatemia does not appear to have a predictive value for the outcome or duration of hospitalization in ICU canine patients. Future longitudinal studies focusing on specific diseases or presentations, as well as the trend of serum phosphate over time as a prognostic indicator, may be warranted to further investigate the clinical significance of phosphate in ICU canine patients.

The low number of hypophosphatemic dogs included in our study can be perceived as a limitation when investigating outcomes and duration of hospitalization. However, this directly reflects the very low prevalence of hypophosphatemia in our ICU canine population. Moreover, the retrospective nature of our study comes with certain limitations, including the exclusion of cases with incomplete data, the challenge of fully accounting for all factors influencing prognosis, and the lack of a marker for disease severity.

## 5 Conclusions

The results of the present study indicate that the prevalence of hypophosphatemia is very low in dogs admitted to the ICU. Additionally, hypophosphatemia does not appear to have prognostic value for the outcome and duration of hospitalization in ICU canine patients. Nonetheless, a multicentric study with a larger population of hypophosphatemic dogs may be warranted to confirm the results of the present study.

## Conflicts of Interest

The authors declare no conflicts of interest.
